# Factors associated with health literacy in rural areas of Central China: structural equation model

**DOI:** 10.1186/s12913-019-4094-1

**Published:** 2019-05-10

**Authors:** Yaofei Xie, Mengdi Ma, Ya’nan Zhang, Xiaodong Tan

**Affiliations:** 1 School of Health Sciences, Wuhan University, No.115 of Donghu Road, Wuhan, 430000 China; 2 Wuhan Blood Center, No.8 of Baofeng One Road, Wuhan, 430000 China

**Keywords:** Health literacy, Factor, Rural area, Structural equation model

## Abstract

**Background:**

Health literacy is a strong predictor of health status. This study develops and tests a structural equation model to explore the factors that are associated with the health literacy level of rural residents in Central China.

**Methods:**

The participants were recruited from a county-level city in Central China (*N* = 1164). Face-to-face interviews were conducted to complete the self-designed questionnaire of each participant. The questionnaire included items for the (1) demographic information, (2) socioeconomic status, and (3) health literacy of the participants. Mplus analyses were performed to evaluate the proposed model.

**Results:**

The final model showed good fit for the data, and both demographic characteristics (i.e., age, BMI, and residence) and socioeconomic status (i.e., monthly income, occupation, and education level) were significantly associated with health literacy level. The effects of these two variables were − 0.277 (*P* < 0.05) and 0.615 (*P* < 0.001), respectively, and the model explained 70.2% of the variance in health literacy.

**Conclusions:**

Health literacy was significantly associated with age, BMI, distance between residence and nearest medical institution, monthly income, occupation, and education level, whereas socioeconomic status was a dominant predictor of health literacy level. Targeting these factors might be helpful in allocating health resources rationally when performing health promotion work.

**Electronic supplementary material:**

The online version of this article (10.1186/s12913-019-4094-1) contains supplementary material, which is available to authorized users.

## Background

Health literacy (HL) refers to the ability of an individual to obtain, understand and use health information to maintain and improve his/her own health [1]. Compared with traditional sociodemographic factors (e.g., age, race, education, income, and employment status), HL is as a stronger predictor of an individual’s health status [2] and is a significant component of his/her health behaviors, health quality, and access to healthcare. HL also shows an inverse relationship with healthcare utilization and expenditure [3] and plays a crucial role in health education and promotion. Therefore, HL has become an important public health concern and has received increasing attention from different population groups, including patients with asthma [4], HIV [5], lower back pain [6], and other health issues [7–10].

Although the impacts of HL have been widely studied, only few have investigated those factors associated with HL. Some studies revealed that the age, region (urban vs. rural), occupation, education, annual household income, and self-reported health status of individuals were associated with their HL [11–13]. Other works reported that those adolescents coming from prestigious schools and raised by highly educated parents tend to have adequate knowledge, skills, and behaviors [14]. As for occupational population, age, education levels, and type of industry show a significant relationship with HL [15].

Previous studies have mostly employed multiple logistic analyses to explore the factors associated with HL. However, given that the structural equation model (SEM) can provide a more comprehensive and flexible approach to data analysis compared with other multivariate statistical models [16], the purpose of this study was to develop and test a hypothesized model that uses SEM to explore those factors associated with HL and to gain a comprehensive picture of HL among the population of a county-level city in Central China.

## Methods

This cross-sectional study employs SEM to examine the relationships among the studied variables for two reasons. First, SEM can simultaneously estimate the structural relationships between multiple independent variables and the dependent variable. Second, SEM shows advantages in identifying the direct, indirect, and total effects among variables [17].

### Sample

**Fig. 1:**
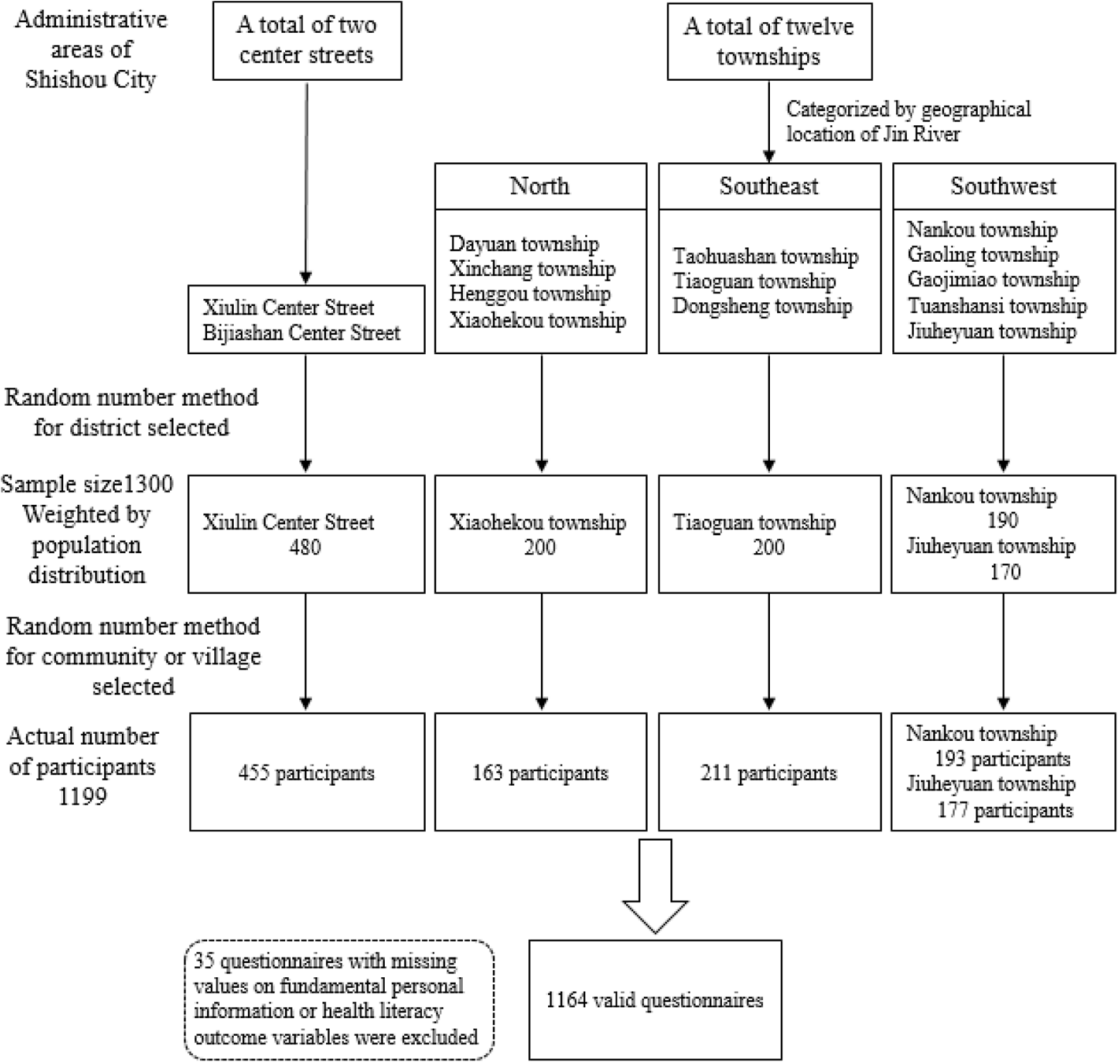
Procedure of multistage sampling

This study was conducted in the city of Shishou, Hubei Province, China on January 2017. A multistage cluster sampling method was adopted for the participant selection. The procedure is illustrated in Fig. 1. Shishou city has 2 center streets and 12 townships within its jurisdiction. Among these townships, four are located north, three are located southeast, and five are located southwest of Jin River that flows through the city. By using the random number method, one central street, one township in the north and southeast parts of the city, and two townships in the southwest part were selected for the sampling. In each selected district, one community or administrative village was randomly selected as an investigation site from which the participants were chosen. The total sample size was set to 1300 or at least 2‰ of the 576.4 thousand people residing in Shishou City. All participants should be aged between 15 years and 79 years. The sample size for each selected district was weighted by the proportion of its population to that of the entire Shishou City. Face-to-face interviews based on a self-designed questionnaire were conducted to collect information from each participant who signed an informed consent. The research team included three PhD candidates and nine master candidates in the School of Health Sciences of Wuhan University and the public health physicians from local health agencies. All these investigators were trained on the research purpose, survey procedure, and other important matters before conducting the survey. Those questionnaires with missing values for important personal information or HL outcome variables were excluded from the analysis.

### Latent variables

Many studies have reported that sociodemographic factors, including gender, age, race, marital status, education level, and income, are associated with one’s HL level [11, 18–20]. Based on these univariate factors, we hypothesize that two latent variables, namely, demographic factor sand socioeconomic status (SES), can be extracted and used to collectively reflect the basic demographic characteristics and SES of the participants, respectively. Therefore, our hypothesized model includes three latent variables, namely, demographic information, SES, and HL.

A three-part self-designed questionnaire was used for the data collection. Six types of demographic information were collected, namely, gender, age, height, weight, marital status, and residence. BMI was calculated based on height and weight. SES comprised three observed variables, namely, monthly income, occupation, and education. HL content (Additional file 1) was assessed based on the “2015 Questionnaire of Health Literacy” scale developed by the Chinese Ministry of Health. This variable was divided into the three dimensions of health knowledge, health behavior, and health skill. Health knowledge refers to one’s understanding of basic and scientific health concepts, such as healthy lifestyle, safe medication, and chronic disease prevention and control. Health behavior mainly includes daily health-related lifestyles, including diet, exercise, smoking, and drinking. Health skill refers to one’s ability to obtain and understand information related to their health and self-rescue ability. All observed variables are summarized in Table 1.

**Table 1:** Assignment of observed variables

Variables	Value
Demographic information	
1.Gender	1 = male, 2 = female
2.Age in years	1 = 15–29, 2 = 30–44, 3 = 45–59, 4 = 60–69, 5 = 70–79
3.BMI	1 = underweight, 2 = healthy weight, 3 = overweight, 4 = obese
4.Marital status	1 = unmarried, 2 = married, 3 = divorce, 4 = widowed
5.Residence: location from nearest medial institutions	1 = < 1 km, 2 = ≤3 km, 3 = ≤5 km, 4 = >5 km
SES	
6.Monthly income	1 = < 1000 RMB [¥], 2 = 1000–2999 RMB [¥], 3 = 3000–4999 RMB [¥], 4 = ≥5000 RMB [¥]
7.Occupation	1 = unemployed, 2 = agricultural worker, 3 = worker, 4 = business personnel, freelancer, 5 = enterprise worker, 6 = institutional staff, 7 = civil Servants
8.Education	1 = primary school or below, 2 = junior high school, 3 = senior high school, 4 = associate bachelor or above
Health literacy	
9.Health knowledge	1 = poor status (correct rate < 80%) 2 = not poor (correct rate ≥ 80%)
10.Health behavior	1 = poor status (correct rate < 80%) 2 = not poor (correct rate ≥ 80%)
11.Health skills	1 = poor status (correct rate < 80%) 2 = not poor (correct rate ≥ 80%)

### Statistical analysis

The descriptive statistics for the demographic variables and HL status were analyzed along with the correlation matrix for all observed variables by using SPSS version 20.0. SEM was performed using Mplus version 7.0 to test the hypotheses. The weighted least squares with mean and variance adjusted method was used for the parameter estimation.

A two-step approach was adopted to test the associations among the variables. First, a confirmatory factor analysis for the measurement model was conducted to examine how well the latent variables can be reflected by the observation variables. Second, the hypothesized SEM was tested to examine the associations among the three latent variables. The overall model fitness was confirmed by using fitness indices to check whether the hypothesized model fits the data well. These goodness-of-fit indices include: maximum likelihood chi-square (*χ*^2^), comparative fit index (*CFI*), Tucker–Lewis index (*TLI*), and root mean square error of approximation (*RMSEA*).

## Results

### Participants and HL status

Among the 1199 participants, 1164 returned valid responses. Among these 1164 participants, 51.80% were males (*n* = 603), with a median age of 47 years inter-quartile range of 25 years, 17.61% had qualified HL (*n* = 205), and 29.72% (*n* = 346), 18.73% (*n* = 218) and 35.22% (*n* = 410) had health knowledge, health behavior, and health skill, respectively. As shown in Table 2, those participants with different genders, age groups, marital status, distances between residence and nearest medical institution, income groups, occupations, and education levels showed significant differences in their rate of qualified HL.

**Table 2:** Participant characteristics by health literacy

Variables	N/%	Health literacy N/%	*χ* ^2^ */ P*
gender			11.686^***^
male	603 (51.80)	84 (13.93)	
female	561 (48.20)	121 (21.57)	
Age (years)			135.154^***^
15~29	248 (21.31)	84 (33.87)	
30~44	256 (21.99)	78 (30.47)	
44~59	426 (36.60)	38 (8.92)	
60~69	151 (12.97)	3 (1.99)	
70~79	83 (7.13)	2 (2.41)	
BMI			3.215
emaciation	100 (8.59)	16 (16.00)	
normal	703 (60.40)	135 (19.20)	
overweight	292 (25.09)	43 (14.73)	
obesity	69 (5.93)	11 (15.94)	
Marital status			32.555^***^
unmarried	197 (16.92)	58 (29.44)	
married	923 (79.30)	144 (15.60)	
divorced and widowed	44 (3.78)	3 (6.82)	
Residences (km)			10.844^*^
< 1	604 (51.89)	97 (16.06)	
≤ 3	376 (32.30)	85 (22.61)	
≤ 5	117 (10.05)	14 (11.97)	
> 5	67 (5.76)	9 (13.43)	
Income (RMB/month)			35.646^***^
< 1000	333 (28.61)	27 (8.11)	
< 3000 h	601 (51.63)	119 (19.80)	
< 5000	171 (14.69)	48 (28.07)	
≥ 5000	59 (5.07)	11 (18.64)	
Occupation			284.675^***^
unemployed	166 (14.26)	27 (16.27)	
farmer or worker	594 (51.03)	37 (6.23)	
businessman and freelancer	160 (13.74)	21 (13.12)	
enterprise staff	34 (2.92)	8 (23.53)	
institution or government staff	210 (18.04)	125 (51.23)	
Education			342.265^***^
Primary school or below	361 (31.01)	5 (1.39)	
Junior high school	433 (37.20)	54 (12.47)	
Senior high school	179 (15.38)	46 (25.70)	
associate bachelor or above	191 (16.41)	100 (52.36)	
Total	1164 (100.00)	205 (17.61)	–

^*^P < 0.05, ^**^P < 0.01, ^***^P < 0.001

The correlation matrix for all observed variables is presented in Table 3. Many of the observed variables showed a significant correlation. Health knowledge, health behavior, and health skill were all highly correlated with age and the observed variables of socio-economic status (*p* < 0.01).

**Table 3:** Correlation matrix for study variables (*N* = 1164)

	1	2	3	4	5	6	7	8	9	10	11
Demographic information											
1.Gender	1										
2.Age	−.100^**^	1									
3.BMI	−.156^**^	.096^**^	1								
4.Marital status	.003	.550^**^	.104^**^	1							
5.Residence	.081^**^	.095^**^	.045	.090^**^	1						
Socioeconomic status											
6.Monthly income	−.073^**^	−.360^**^	.003	−.184^**^	−.016	1					
7.Occupation	.023	−.279^**^	−.028^*^	.015	−.074^**^	.293^**^	1				
8.Education	−.057^*^	−.556^**^	−.055^*^	−.230^**^	−.132^**^	.369^**^	.688^**^	1			
Health literacy											
9.Health knowledge	.057^*^	−.375^**^	−.066^*^	−.183^**^	−.006	.187^**^	.347^**^	.435^**^	1		
10.Health behavior	.061^*^	−.257^**^	−.067	−.095^**^	.007	.136^**^	.336^**^	.374^**^	.372^**^	1	
11.Health skill	−.053^*^	−.213^**^	−.018	−.104^**^	−.090^**^	.139^**^	.255^**^	.282^**^	.288^**^	.301^**^	1

*P < 0.05

**P < 0.01

### Measurement model

**Fig. 2:**
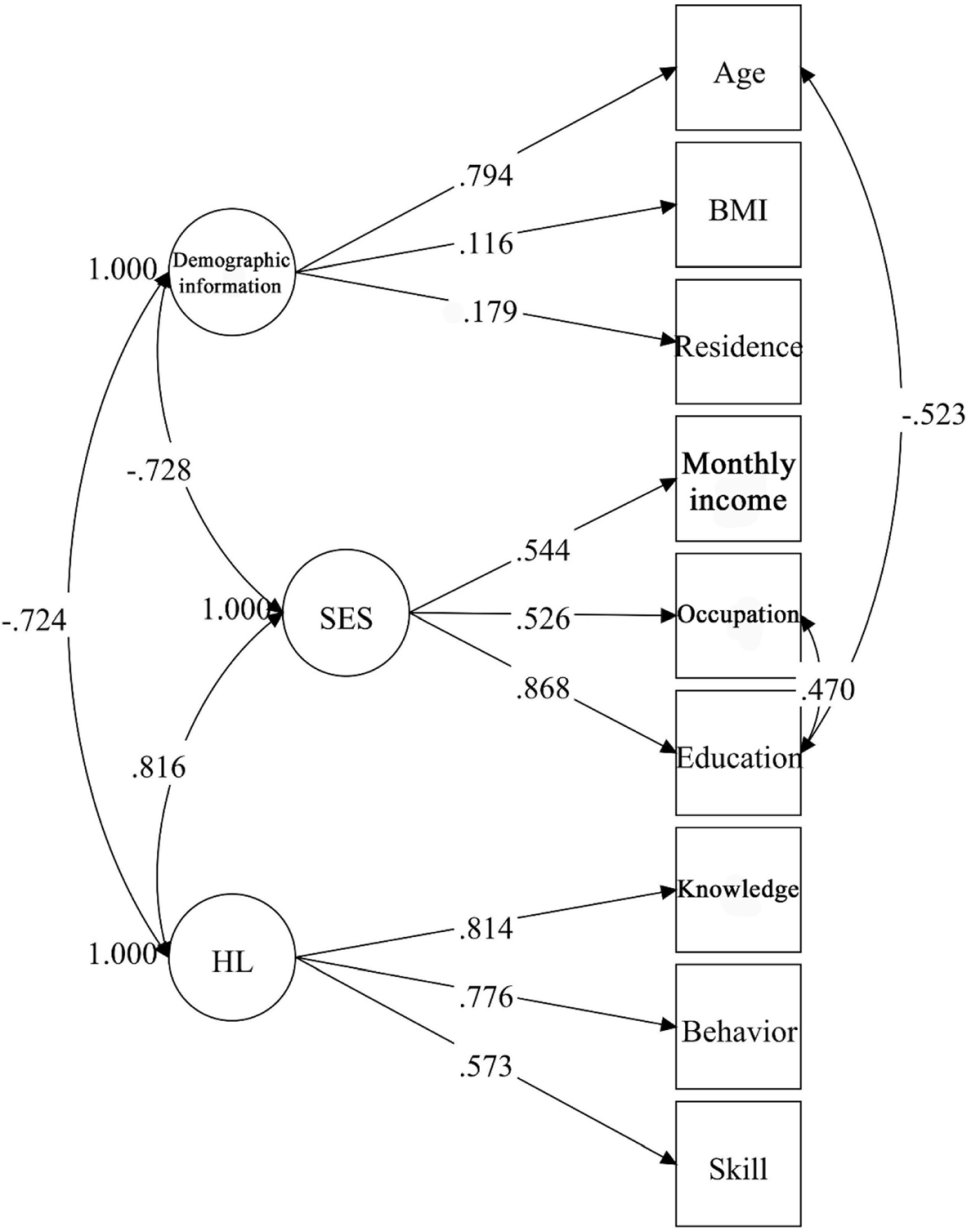
Final measurement model of the observed variables. Three latent variables and nine manifest variables are connected by significant paths. The numbers on the straight arrows indicate the path coefficients. Every pair of latent variables is connected by bidirectional arrow curves, and the numbers on the lines indicate the correlation coefficients. The variances are set to 1.000 during the model estimation

Demographic information, SES, and HL were specified in the measurement model. To determine the most parsimonious model, the insignificant parameters and collinear observed variables were deleted from the initial measurement model based on the critical ratios and regression coefficients. After this modification, demographic information, SES, and HL were assigned three, two, and three observed variables, respectively. (Fig. 2).

Demographic information, SES, and HL had loadings of 0.18–0.79, 0.54–0.87, and 0.57–0.81, respectively. All these factor loadings were significant (*p* < 0.01).

Three latent variables from the measurement model were significantly intercorrelated. HL showed the strongest relationship with SES (*r* = 0.82, *p* < 0.01) and demonstrated a strong association with demographic information (*r* = − 0.72, *p* < 0.01). Meanwhile, SES showed a strong association with demographic information (*r* = − 0.73, *p* < 0.01), and the measurement model demonstrated an acceptable fit to the data: *χ*^2^ = 106.608, *df* = 17, *p* < 0.001, *CFI* = 0.946, *TLI* = 0.912, *RMSEA* = 0.067 (95% *CI* = 0.055–0.080).

### Structural equation model

**Fig. 3:**
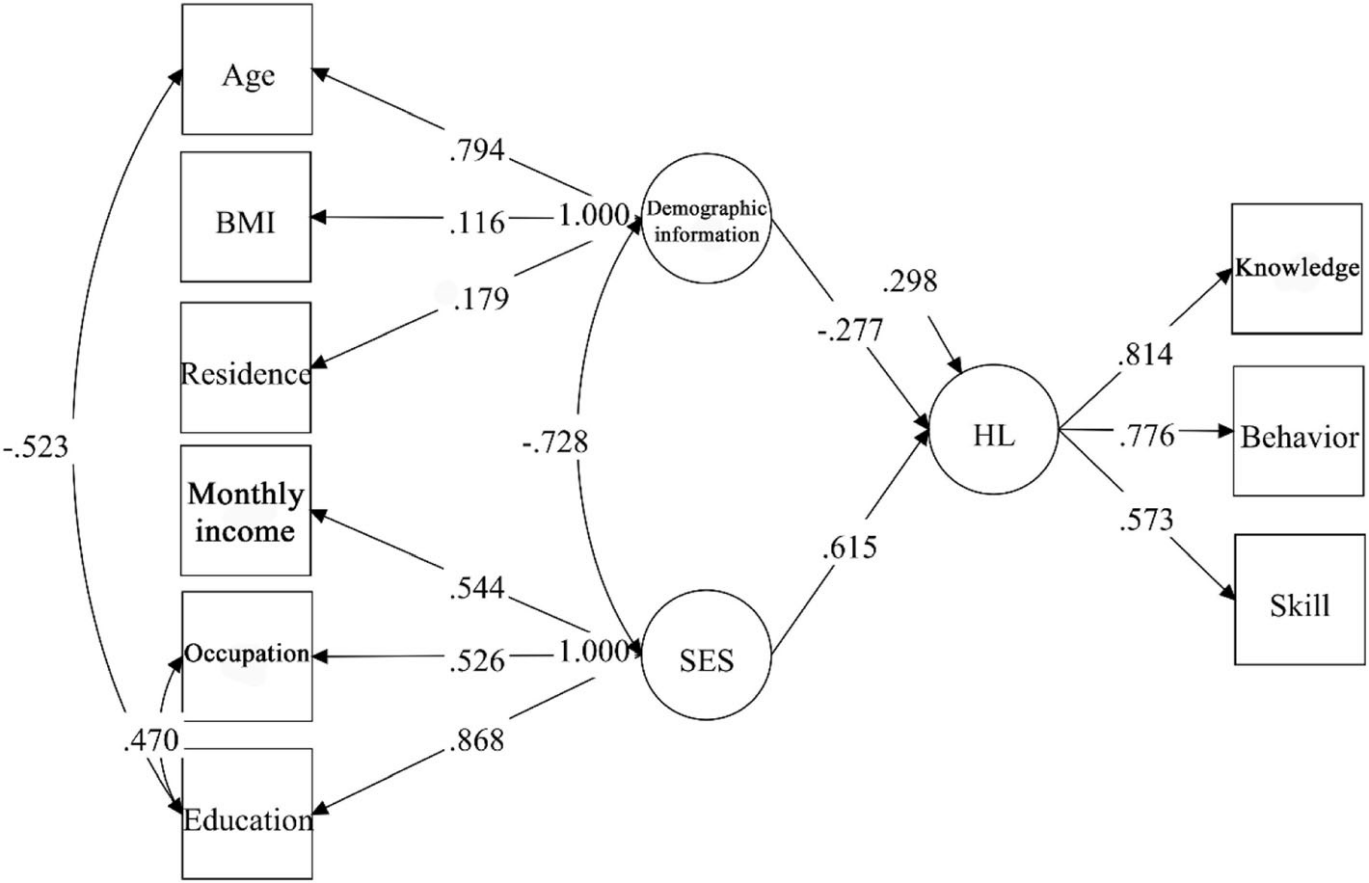
Structural equation model of demographic information, socioeconomic status, and HL. The relationships between the three latent variables and their corresponding manifest variables are presented, and the standardized coefficients and residuals are included in the model. Demographic information and SES are significantly correlated with a correlation coefficient of − 0.728. HL is significantly associated with demographic information and SES with path coefficients of − 0.277 and 0.615, respectively

A structural equation model was designed based on the measurement model. Demographic information (*r* = − 0.28, *p* < 0.05) and SES (*r* = 0.615, *p* < 0.01) had direct paths to HL and were correlated. The developed structural equation model was the most parsimonious model in which all the parameters were significant (*p* < 0.05 or *p* < 0.01). This model also showed a good fit to the data: *χ*^2^ = 106.608, *df* = 17, *p* < 0.001, *CFI* = 0.946, *TLI* = 0.912, *RMSEA* = 0.067 (95% *CI* = 0.055–0.080). (Fig. 3).

Demographic information and SES were both significantly and directly associated with HL. According to the structural equation model, the identified predictors explained 70.2% of the variance in HL. Table 4 illustrates the direct associations of all observed variables with HL.

**Table 4:** Standardized effects of observed variables on health literacy

Variables	Effects
Demographic information	
Age	−0.499
BMI	−0.032
Residence	0.050
Socioeconomic status	
Monthly income	0.335
Occupation	0.574
Education	0.801

## Discussion

This study is the first to examine the structural interrelationship of the three facets of HL with possible associated factors among the population of Central China. HL showed a significant relationship with both demographic information and SES, and these two predictors could altogether explain 70.2% of the variance in HL. The direct pathway of the model highlighted SES as a dominant predictor of HL level.

Demographic information showed a significantly negative relationship with the three facets of HL. The paths indicated that older participants with higher BMI and were residing further away from the nearest medial institution were more likely to have a lower HL level. The elderly population usually had less opportunities and approaches to access to health education. Overall, the Chinese elderly population had limited access to education during their youth, which coincided with the findings of some previous studies [2, 11, 12, 21]. Few studies have highlighted a relationship between BMI and HL. For instance, in a population cohort study among men in South Australia revealed an association between low HL and obesity [22], while a study among dialysis patients showed that HL was not a statistically significant predictor of BMI [23]. Meanwhile, in this study, the relationship of BMI and HL was statistically significant but not particularly strong. This result may be explained by the close relationship between one’s BMI and lifestyle and the fact that those individuals with higher HL were more likely to adopt healthy eating, exercise, and sleep behaviors [24]. Residence from the nearest medial institution was also associated with health education opportunities.

The association among income, occupation and HL has been highlighted in previous studies [2, 11, 12, 25, 26], whose findings are consistent with those of this work, specifically the result highlighting monthly income and occupation as strong predictors of HL. The association between education level and HL level has also been reported in many studies [13, 15, 27–30]. Similar to the findings of this work, those individuals with higher education level were reported to have a higher HL level. Because having higher access to education could improve one’s ability to read, analyze, and judge information. In other words, people with better education had easier access to health knowledge and skills.

### Limitations

This study has several limitations. First, the results are based on a cross-sectional sample, and are therefore unable to highlight the direction of causality of some factors. Second, the model results were based on classificatory data, some of which were converted from continuous variables, and this data processing might result in information loss. Third, this study only focused on the residents of a single county in China, thereby restricting the generalizability of its findings. To address these issues, the model should be validated by using multiple samples, and future studies should focus on the residents of other provinces of China verify the findings and to better understand the relationship between HL and its associated factors.

## Conclusions

The findings of this work indicate that demographic information and SES are associated with the HL level of the residents of a county-level city in Central China. Older people, with higher BMI, residing far away from medical institutions, earning a lower monthly income, and having lower education levels were more likely to have a lower level of HL. Occupation also plays a significant role in predicting HL. Accordingly, the aging, obese, and undereducated population as well as those residents living far away from medical institutions warrant more attention when performing health education or promotion work. Health resources should also be allocated rationally to different occupational groups to enhance their utilization efficiency.

## Additional file

Additional file 1: Health Literacy Survey Part. This additional file is the health literacy survey part of the questionnaire, it includes four types, a total of fifty six questions about health literacy. And these questions are used to test the level of health knowledge, health behavior and health skills of the participant. (DOC 98 kb)

## Abbreviations

BMI: Body mass index
CFI: Comparative fit index
CI: Confidence interval
HIV: Human immunodeficiency virus
HL: Health literacy
RMSEA: Root mean square error of approximation
SEM: Structural equation model
SES: Socioeconomic status
TLI: Tucker-Lewis index
