# Supplementary material for: Factors associated with health literacy in rural areas of Central China: structural equation model
Source: BMC Health Serv Res. 2019 May 10;19:300. doi: 10.1186/s12913-019-4094-1 (PMC6509858; doi:10.1186/s12913-019-4094-1)
Supplement: Supplementary file 1 — Health Literacy Survey Part. This additional file is the health literacy survey part of the questionnaire, it includes four types, a total of fifty six questions about health literacy. And these questions are used to test the level of health knowledge, health behavior and health skills of the participant. (DOC 98 kb) [file 12913_2019_4094_MOESM1_ESM.doc]

Health Literacy Survey Part

**A True or False questions (please choose "①" if you think the statement is correct, otherwise choose "②".)**

**A01.** The best way to prevent flu is to take antibiotics (anti-inflammatory drugs).

① Right ② Wrong

**A02.** Health foods are not medicines, nor can they replace medicines.

① Right ② Wrong

**A03.** Infusion is effective and fast, so you should first choose infusion after illness.

① Right ② Wrong

**A04.** Fruits and vegetables have similar nutrients, so you can replace eating vegetables into eating fruits.

① Right ② Wrong

**A05.** Normal people's body temperature can fluctuate up and down in one day, but the fluctuation range generally does not exceed 1 °C.

① Right ② Wrong

**A06.** Children and adolescents may also experience depression.

① Right ② Wrong

**A07.** Residents can get free health knowledge at community health service centers (stations) and township health centers (village clinics).

① Right ② Wrong

**A08.** "Long illness makes the patient a good doctor", patients with chronic diseases can adjust the treatment plan according to their own feelings.

① Right ② Wrong

**A09.** For health problems and diseases found during a health checkup, if there are no symptoms, no measures need to be taken immediately.

① Right ② Wrong

**B Single choice questions (Each question has only one correct choice. If you don't know, please select "④".)**

**B01.** Concerning the concept of health, the complete statement is:

① Health means being strong and disease-free.

② Health means good psychological quality and strong physique.

③ Health is not only the absence of disease, but also the sound state of physical, psychological and social adaptation.

④ I don't know.

**B02.** Which of the following ways can hepatitis B be transmitted to others?

① Work, eat, and swim with the patient or infected person

② Sexual behavior, blood transfusion, mother-to-child transmission.

③ Talk, shake hands, and hug with the patient or infected person.

④ I don't know.

**B03**. Concerning self-blood pressure measurement, which statement is wrong?

① Self-measured blood pressure has reference value for the diagnosis of hypertension.

② Regular self-monitoring of blood pressure in patients with hypertension can provide a basis for doctors to formulate treatment plans and evaluate the therapeutic effect.

③ As long as the blood pressure of hypertension patients is stable, they don’t have to go to the clinic regularly for follow-up treatment.

④ I don't know.

**B04.** Concerning the danger of smoking, which statement is wrong?

① Tobacco dependence is a chronic addictive disease.

② Smoking can cause many kinds of chronic diseases.

③ Low tar cigarettes are less harmful than ordinary cigarettes.

④ I don't know.

**B05.** Which statements is not an early warning sign of cancer?

① Abnormal lump in the body.

② Unclear hematochezia.

③ Gain weight.

④ I don't know.

**B06.** After the occurrence of gas poisoning, how should the rescuers firstly deal with the people with gas poisoning?

① Give the patient water to drink.

② Move the patient to a ventilated place.

③ Call 120 for hospital treatment.

④ I don't know.

**B07.** Concerning the treatment of tuberculosis patients, which statement is correct?

① No preferential policies.

② Free anti-tuberculosis drugs provided by the state.

③ Free hospitalization.

④ I don't know.

**B08.** When engaging in toxic and hazardous operations, the staffs should:

① Wear overalls.

② Wear safety helmet.

③ Use personal occupational disease protection equipment.

④ I don't know.

**B09.** The main hazard of iodine deficiency is:

① Suffering from SARS.

② Influencing the developments of intelligence and growth.

③ Causing hypertension.

④ I don’t know.

**B10.** During vigorous activities, water will be lost due to sweating. In this case, it's better to drink:

① Boiled water.

② Sugary drinks.

③ Dilute brine.

④ I don’t know.

**B11.** Concerning the National basic public health service, which statement is wrong?

① Mainly conducted in large hospitals.

② Conducted in primary medical and health institutions.

③ People can enjoy it free of charge.

④ I don’t know.

**B12.** In which of the following cases, vaccination of children should be suspended?

① Crying.

② Have a cold/fever.

③ Within half an hour after meals.

④ I don’t know.

**B13.** When you have fever symptoms, the right thing to do is:

① Seeking a doctor in time

② According to past experiences, take antipyretics by yourself.

③ Observing the situation for a while.

④ I don’t know.

**B14.** When patients suffer from adverse reactions after taking medicine according to doctors’ treatment plans, the right thing to do is?

① You stop taking the medicine.

② Seeking a doctor.

③ Taking medicines continually.

④ I don’t know.

**B15.** If you want to know whether a medical institution is legal, which way can you judge it by?

① The scale of hospital.

② Consulting the local health bureau or visiting the website of the Health Bureau.

③ Medical facility conditions.

④ I don’t know.

**B16.** If a strong infectious disease occurs in a certain place, the right thing to do is?

① This disease has nothing to do with me, and I don’t care about it.

② I would be concerned about the epidemic if I were a local.

③ Whether local or not, we need to pay attention to the change of epidemic situation.

④ I don’t know.

**B17.** Warning map
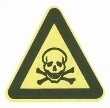
 means:

① The place is prone to fire.

② Explosives are present in an area of the site and are not allowed to approach.

③ This substance is toxic or there are toxic substances in the place.

④ I don’t know.

**B18.** The phone number of the national free health hotline is:

① 12302

② 120

③ 12320

④ I don’t know.

**B19.** Concerning seeking medical treatment, which statement is wrong?

① Tell the doctor as detailed as possible about the illness.

② If there are previous medical records, examination results, etc., it is best to carry them with you.

③ In order to make the doctor pay attention to it, we can describe the condition more seriously than it actually is.

④ I don’t know.

**B20.** If “OTC” is printed on a drug label, the drug shall be:

① A prescription drug，which must be prescribed by a doctor before it can be purchased.

② A Over-the-counter drug, which can be purchased without a doctor's prescription.

③ Health products.

④ I don’t know.

**B21.** Concerning window ventilation, which statement is wrong?

① Avoid colds by opening fewer or no windows in winter.

② Window ventilation can dilute bacteria and viruses in indoor air.

③ Opening windows and ventilation can let sunlight into the room, killing a variety of bacteria and viruses.

④ I don’t know.

**B22.** When measuring body temperature with a vitreous thermometer, the correct reading method is:

① Hold the mercury end of the thermometer and read it horizontally.

② Hold the glass end of the thermometer and read it vertically.

③ Hold the glass end of the thermometer and read it horizontally.

④ I don’t know.

**B23.** The normal number of pulses in adults is:

① 30-50 times/min.

② 60-100 times/min.

③ 100-120 times/min.

④ I don’t know.

**B24.** How many times of pregnancy checkups should woman have from pregnancy to childbirth?

① 3

② 5

③ 7

④ I don’t know.

**B25.** When blisters appear caused by mild scalds of the skin, which statement is right?

① Break the blisters, so that the recovery will be quick.

② Bubbles don’t need to be pricked if they’re small, but must be pricked if they’re large.

③ Do not prick blisters to avoid infection.

④ I don’t know.

**B26.** In case of fire, the correct escape method is:

① Hold your head in your hands, or wrap your head in your clothes, and rush out of the fire.

② Sprinkle water on your head and body, or wrap your body in a wet blanket, and rush out of the fire.

③ Beating at the flames with clothes, while evacuating from the fire.

④ I don’t know.

**C** **Multiple choice questions (Each question has two or more correct choices. If you don't know, please select ⑤.)**

**C01.** Which of the following are true about the ways to promote mental health?

① Keep an optimistic attitude towards life

② Set target within the scope of your own ability

③ Establish good interpersonal relations, and actively participate in social activities

④ Solve the worry through smoking, drinking

⑤ I don't know

**C02.** Which of the following are true about seeking medical advice?

① Not all diseases can be cured

② As long as going to hospital, the health status will be improved

③ A hospital is the place of healing, it is the hospital's responsibility not to cure the disease

④ Sickness and death are natural, rationally treated the diagnosis and treatment results

⑤ I don't know

**C03**. Which of the following are true about liver?

① Secrete bile

② Detoxification function

③ Most important digestive organ

④ The liver has one on each side

⑤ I don't know

**C04.** What should parents do while children appear fever, rash and other symptoms?

① Go to the hospital in time

② Stop going to kindergarten

③ Inform the teacher of the kindergarten in time

④ Let the child go to kindergarten as usual

⑤ I don't know

**C05.** Which of the following statement are true?

① Treating osteoporosis in old age is too late

② Osteoporosis is a normal physiological phenomenon of aging

③ Middle-aged and elderly people can reduce bone loss through drinking milk

④ More exercise can prevent osteoporosis

⑤ I don't know

**C06.** What information should be noted on the package while buying packaged food?

① Production date

② Expiration date

③ Nutrition facts

④ Manufacturer

⑤ I don't know

**C07.** What should be done if dead or sick animals are found?

① no killing, no processing

② Not for sale, not for transport

③ don't eat

④ eating after cooking thoroughly

⑤ I don't know

**C08.** What measures should be taken for patients suffering from respiratory or cardiac arrest?

① Artificial respiration

② Chest compressions

③ Call the emergency number

④ Give hypertension treatment drugs

⑤ I don't know

**C09.** The benefits of eating tofu, soy milk and other soy products:

① Good for health

② Good for patients with cardiovascular diseases

③ Increase protein intake

④ Prevent excessive consumption of meat

⑤ I don't know

**C10.** The health benefits of exercise:

① Maintain a reasonable weight

② Prevent chronic diseases

③ Reduce psychological pressure

④ Improve sleep quality

⑤ I don't know

**C11.** A newspaper said that any diabetic can be completely cured by taking a certain hypoglycemic product. Which of the following statements are true after seeing this information?

① The news is not believable

② This news is good, hurry to tell diabetes friends

③ Consult and verify it with the community doctor

④ Hurry to buy

⑤ I don't know

**C12.** When coughing or sneezing, the correct ways are:

① Use hands to cover your nose and mouth directly

② Use a handkerchief or tissue to cover mouth and nose

③ Cover nose and mouth with elbow

④ Don't cover mouth and nose

⑤ I don't know

**C13.** Which of the following statement about hospitalization are true?

① The longer length of hospitalization, the better effect of the treatment

② Treatment effect and length of hospitalization is not necessarily related

③ The length of hospitalization depends on the patient's condition

④ The short length of hospitalization is a sign of doctors' irresponsibility

⑤ I don't know

**C14.** Which of the following statements about medical treatment were correct?

① One should go to a tertiary-grade A class hospital when get sick

② One should try to choose nearby community hospital for diagnosis and treatment at first and

go to a tertiary-grade A class hospital when necessary

③ One should return to the community for management during later rehabilitation treatment

④ One should go to a tertiary-grade A class hospital during later rehabilitation treatment

⑤ I don't know

**C15.** Which of the following benefits of breastfeeding for infants were correct?

① Breastfeeding can lead to less babies sick

② Breast milk is the best natural food for babies

③ Infant formula is more nutritious than breast milk

④ Breastfeeding is conducive to the psychological development of infants

⑤ I don't know

**C16.** When keeping pesticides, attention should be paid to:

① Pesticides should be kept in a fixed, safe place

② Pesticides cannot be put together with food

③ If the hand is contaminated with pesticides, washing hand is not necessary as long as the skin is damaged.

④ Pesticide should be kept in the place where children can not touch

⑤ I don't know

**C17.** Out during thunderstorms, which of the following statements are correct?

① Hiding under the big tree

② Staying away from the high voltage line

③ Avoid using cell phone

④ Standing in the highlands

⑤ I don't know

**D Situational questions (Please read the material first and then answer the questions. There is only one correct answer for a single choice question and two or more correct answers for a multiple choice question.** **If you do not know, single choice please choose ④, multiple choice please choose ⑤.)**

BMI refers to body mass index (BMI), which is a commonly used international standard to measure body fat and thinness as well as health. This is calculated by dividing weight (kg) by height (m) squared, or BMI= weight/height^2^ (kg /m^2^). For Chinese adults, BMI<18.5 means underweight, 18.5≤BMI<24 means normal weight, 24≤BMI<28 means overweight, and BMI≥28 means obesity.

**D01**. Mr. Li, 45 years old, 170 centimeters in height, 160 pounds in weight, how should his BMI be calculated? (Single choice)

① (80)2 /170=37.6 ② 80/(1.7)2 =27.7 ③ 160/(1.7)2=55.4 ④ I don't know

**D02.** With reference to the body mass index of Chinese adults, Mr. Li belongs to: (Single choice)

① obesity ② normal weight ③ overweight ④ I don't know

**D03.** What can Mr. Li do to control his weight? (Multiple choice)

① Do not eat staple food

② Exercise for at least half an hour every day

③ Reduce oil intake

④ Only eat fruits and vegetables

⑤ I don't know

**D04.** Which of the following diseases is Mr. Li prone to? (Single choice)

① hypertension ② osteoporosis ③gastric ulcer ④ I don't know
